# C-Reactive Protein and Breast Cancer: New Insights from Old Molecule

**DOI:** 10.1155/2015/145647

**Published:** 2015-11-26

**Authors:** Shilpa Balaji Asegaonkar, Balaji Narayanrao Asegaonkar, Unmesh Vidyadhar Takalkar, Suresh Advani, Anand Pandurang Thorat

**Affiliations:** ^1^Department of Biochemistry, Government Medical College, Aurangabad 431001, India; ^2^United CIIGMA Hospital, Aurangabad, India

## Abstract

Recently an association between breast cancer and inflammation has emerged as the seventh hallmark of cancer. Chronic inflammation is a key contributor in the development and progression of carcinogenesis. Inflammatory pathways play an important role in the causation of breast cancer. C-reactive protein (CRP) an acute-phase reactant inflammatory protein is synthesized in hepatocytes in response to cytokines that are released from leucocytes within the tumor microenvironment. Several epidemiological studies appraised an association of CRP with breast cancer risk with inconsistent findings. Elevated levels at the time of diagnosis of breast cancer indicate aggressiveness of the tumor. CRP is also a well-established independent prognostic marker. Breast cancer survivors with the state of chronic inflammation are at risk of recurrence and metabolic disturbances. CRP lowering agents along with chemotherapeutic drugs will improve the survival of breast cancer patients. Also, it is a risk predictor for subsequent cardiotoxicity in patients receiving chemotherapy. The present review is aimed at elucidating the role of C-reactive protein, as an inflammatory risk marker and prognostic predictor of breast cancer. It also focuses on conflicting views on the role of CRP in breast cancer and its impact on therapeutic interventions.

## 1. Background

Worldwide breast cancer is the most frequently diagnosed cancer among women and most common cancer-related death worldwide. According to data reported in 2012, about 1.7 million women were diagnosed with breast cancer and it was the most common cause of cancer-related death (522000 deaths in 2012) [1]. Its prevalence is rising at an alarming rate with the modernization of lifestyle, altered fertility pattern, and improved socioeconomic status, imposing an enormous economic burden on health care system. Hence, strategies for prevention and control of breast cancer are of the utmost importance in the field of medical research.

## 2. Chronic Inflammation: Key Contributor to Carcinogenesis

Several etiological factors like age, environmental and genetic factors, endogenous and exogenous endocrine factors have been implicated in the pathogenesis of breast cancer [2]. Recently the association between breast cancer and inflammation has been suggested as the seventh hallmark of cancer [3]. Rudoff Virchow in 1863 observed infiltration of leucocytes in the malignant tissues and proposed the site of chronic inflammation as the origin of cancer. For the first time, he linked inflammation with carcinogenesis [4]. Since then, a number of researchers studied the role of inflammation in the different areas of cancer. Some solid tumors originate at the site of chronic inflammation and some induce inflammatory microenvironment in the tumor [5]. Accumulating evidence suggests the mechanistic connection between inflammation and cancer, which is widely accepted nowadays.

State of chronic low-grade inflammation predisposes a person to cancer by building up an inflammatory microenvironment. Histologically, evidence of obvious inflammation is seen rarely in breast cancer. But inflammatory component is there in the microenvironment of tumor cells, which contain white blood cells, macrophages with cytokines, and chemokine, as principal mediators of inflammation. This cancer-related inflammation facilitates promotion and progression of tumor growth [6]. Chronic inflammation is a key contributor in the development and progression of carcinogenesis. But sparse data and studies are available about the association of inflammatory markers and breast neoplasms.

The present review is aimed at elucidating the role of C-reactive protein, an inflammatory risk marker and prognostic predictor of breast cancer as well. Also we discussed molecular biology, functions, and limitations of CRP molecule.

## 3. C-Reactive Protein: Old Molecule with Novel Insights

C-reactive protein is a classical acute phase reactant protein from pentraxin family. Tillett and Franscis discovered CRP in 1930 and it was named because of its high binding affinity to C-polysaccharide of* Streptococcus pneumoniae*. It is synthesized by hepatocytes in response to inflammation, trauma, and tissue damage. Also a moderate rise in CRP levels is seen in chronic inflammatory states. CRP has a cyclic pentameric structure with calcium-dependent ligand binding. Five identical noncovalently associated protomers are situated symmetrically around a central pore. Each protomer consists of 206 amino acids residues with ligand binding site having a pocket with two ions of calcium. Calcium ions are required for ligand binding and stability of the molecule CRP [7].

CRP is a prototype of short pentraxin, which represents a systemic response to local inflammation. In human beings, serum amyloid protein is also an additional component of short pentraxin [8]. CRP is present in plasma only in pentameric isoform. Gene for CRP is located on the long arm of chromosome 1 (1q21–q23) and synthesis of CRP is under the transcriptional control of cytokines and transcription factors. Interleukin-6 (IL-6) is the main inducer of CRP gene. Trans-acting variants play an essential role in the regulation of CRP gene. Polymorphisms in and outside CRP locus are associated with CRP in circulation. Outside loci are leptin receptors (LEPR), IL-6 receptors, hepatocytes nuclear factor 1 A, and apolipoprotein E locus (APOE) [9].

CRP is raised in circulation in response to acute inflammation, infection, and tissue damage. The rise in CRP levels is proportional to degree of tissue damage. Its plasma half-life is 19 hours and is catabolized by hepatocytes. Since past decade, with the introduction of high sensitivity assay, it has been evolved as emerging nontraditional novel cardiovascular risk marker because of its confirmed role in initiation and progression of atherosclerosis. It is widely used not only for risk stratification of cardiovascular diseases but also as a predictor of type 2 diabetes mellitus, hypertension, metabolic syndrome, pregnancy-induced hypertension, and various types of cancers. It is a sensitive but nonspecific marker of acute and chronic inflammatory conditions like infections, rheumatoid arthritis, and chronic obstructive pulmonary disease. As its concentration is raised in large amount in bacterial infection, it is also useful in differentiating between bacterial and viral infections [6].

Various immunoturbidimetric, nephelometric, and enzyme linked immunosorbent assay methods are available for estimation of serum CRP with detection range of 3 to 8 mg/L. Commercial kits for assay of high sensitivity C-reactive protein (hsCRP) based on immunoturbidimetric, immunonephelometric, and chemiluminescence methods are available with lower detection limit of 0.3 mg/L. Estimation of serum hsCRP is simple, cheap investigation with no diurnal variation. Serum CRP is stable and samples can be stored for long periods at and below −20°C [7]. As it is nonspecific inflammatory marker, its potential clinical utility is still questionable.

## 4. CRP: A Surrogate Marker of Inflammation as Risk Marker of Breast Cancer

Inflammatory pathways play an important role in the causation of breast cancer. There is a bidirectional link between chronic inflammation and carcinogenesis: tumor originates and progresses at the site of chronic inflammation while tumor cells attract immune cells and promote the production of cytokines and chemokine creating tumor microenvironment. Hence, cancer is associated with the persistent inflammatory state. There is a vicious cycle and complex interplay between cancer and inflammation [3]. So researchers proposed that serum CRP could be a marker of increased risk for breast cancer.

Estimation of CRP can be looked at as a simple, cost-effective, easily available screening test to assess future risk of breast cancer.

Several epidemiological studies appraised association of CRP with breast cancer risk with inconsistent data findings. So Guo and associates conducted a meta-analysis of 8 cohort and 7 case-control studies to assess the role of CRP in predicting breast cancer risk. Out of 15 studies, two studies had shown negative, statistically insignificant association between one unit change in ln CRP and breast cancer. The remaining studies observed positive association, among which four reported statistically significant positive association. Combined OR per natural unit change in CRP for breast cancer was 1.16 (95% CI: 1.06–1.27) with moderate heterogeneity. They also observed a strong association in retrospective case-control studies compared to cohort studies. Elevated CRP levels significantly increased a risk of breast cancer among postmenopausal women (OR = 1.08, 95% CI: 1.00–1.16) but not in premenopausal breast cancer (OR = 1.08, 95% CI: 0.91–1.28). In postmenopausal women, overweight and obesity favor a low-grade chronic inflammatory state that predisposes to breast cancer. Serum hsCRP, a sensitive inflammatory marker, had a stronger association than common CRP. Overall analysis of this meta-analysis reported that a natural log unit rise in CRP level results in 16% increase in breast cancer condition [10]. Meta-analysis of prospective cohort studies reported an association between CRP and risk of various types of cancers, strong with lung cancer and weak with breast, prostate, and colorectal cancers supporting role of chronic inflammation in carcinogenesis [11].

One of the prospective studies of risk of breast cancer among nondiabetic women observed significantly increased risk in women with hsCRP more than 3 mg/L versus those women with less than 1 mg/L concentration (HR 1.80, 95% CI+ 1.03–3.15) [12]. Also in one large population-based cohort of 19,437 Chinese Kailuan females, hsCRP was significantly associated with risk of breast cancer in younger women below age of 50 with HR 2.76, 95% CI = 1.18–6.48 [13]. Hong et al. reported 1.65-fold increased risk of breast cancer (OR 1.65, CI 95% 1.12–2.42) among women in the highest quartile of CRP. They also found a positive association in postmenopausal overweight women. Among hormone receptor positive and HER2 negative women, CRP levels were elevated. They linked chronic low-grade inflammation with obesity and breast cancer [14]. In E3N cohort study, overall no association was observed between CRP levels and breast cancer risk, but significant association of CRP levels was found with body mass index, waist circumference, and waist : hip ratio [15]. These studies suggested linkage of chronic low-grade inflammation with abdominal obesity and breast cancer.

Controversial data has been obtained in several studies assessing the role of CRP as a risk marker. In Women's Health Study, serum CRP levels of 27919 healthy women were assayed and this cohort was followed up for 10 years. 892 women developed invasive breast cancer, which was statistically nonsignificant finding. But women with body mass index above 25 Kg/m^2^ and history of past smoking were found to have a significant association between CRP and risk of breast cancer. On multivariate analysis, there was no significant association between CRP and other risk factors and characteristics of breast cancer. So the results of this prospective study concluded that CRP is not associated with risk of breast cancer in apparently healthy women [16]. Among overweight and obese subjects, expanded adipose tissue might release proinflammatory cytokines, which stimulate hepatic CRP synthesis. Il'yasova et al. in their prospective study reported no significant association between CRP and risk of breast cancer [17]. Similar findings were documented in the subsequent prospective study from Greece population [18].

Wang and Sun in their systematic review reported no strong evidence between CRP levels and risk of breast cancer [8]. Some researchers studied an association of anti-inflammatory drugs like NSAIDs and statins with the risk of breast cancer with inconsistent findings [19, 20]. Several Mendelian randomization studies evaluated the association of polymorphism with the risk of different types of cancer [21]. Rotterdam study reported no association of CRP polymorphism and cancer except for lung cancer. Heikkilä and colleagues also concluded that raised CRP levels are unlikely to have a causal role in the carcinogenesis [22].

The exact role of CRP in the pathogenesis of carcinogenesis is uncertain, but evidence from numerous prospective and case-control studies has supported a role of CRP in different aspects of breast cancer. Identification of the role of the molecular pathway in the causation of breast cancer and implementation of anti-inflammatory strategy by therapeutic and lifestyle intervention is important in future to prevent and control incidence of breast cancer.

## 5. CRP as a Prognostic Predictor for Breast Cancer

Numerous prospective epidemiological studies have observed the association of CRP at the time of diagnosis of breast cancer with the prognosis of the disease. CRP is synthesized in hepatocytes in response to cytokines, particularly IL-6, released from leucocytes within the tumor microenvironment. IL-6 also helps in binding CRP to phospholipids on tumor cells that results in activation of classic C1q complement pathway. Here it acts as opsonin leading to tumor cell lysis [23]. State of persistent low-grade inflammation in cancer is associated with the progression of disease and poor outcome. So predictive ability for the outcome of the patients with breast cancer can be improved with the addition of CRP to other prognostic factors.

Moon and associates studied the molecular link between sphingosine-1-phosphate (S1P) and CRP during the invasive process of breast epithelial cells using a xenograft mice tumor model and revealed the molecular basis of S1p-induced transcriptional activation of CRP and its functional significance of invasive phonotype of human breast epithelial cells in an inflammatory environment. S1P upregulates expression of CRP which in turn triggers transcriptional activation of matrix metalloproteinase-9 through reactive oxygen species, calcium ions, and c-fos leading to breast cell invasion [24]. Allin et al. in the prospective study examined the prognostic value of baseline plasma CRP at the time of diagnosis with overall survival (OS), disease-free survival (DFS), death from breast cancer, and recurrence of breast cancer. Among a cohort of 2910 Danish women with breast cancer, OS and DFS were less in subjects with high CRP levels irrespective of the presence of distant metastasis and hormone receptor status. Cumulative incidence of death due to breast cancer and recurrence was highest among women in high quarantine of CRP, but the incidence of recurrence was not increasing in a stepwise manner with rising levels of CRP. Women with CRP above 95% percentile that means above 16.4 mg/L at the time of diagnosis had 3.5-fold increased risk of reduced OS. The study also reported a very strong association of raised CRP with decreased OS in Human Epidermal Receptor 2 (HER 2) positive women [25]. Pierce et al. observed increased CRP levels after 2 and half years of diagnosis in 700 women, which was associated with reduced DFS and OS. These findings suggested the predictive role of CRP in short-term as well as long-term prognosis [26]. Han et al. compared the prognostic role of CRP in their meta-analysis study. They included 10 studies (*n* = 4502) and compared OS, cancer-specific survival, and DFS in patients with raised and low level of CRP. The pooled hazard ratios (HR) for OS and DFS were significant at 1.62 and 1.81, respectively, and 2.08 for cancer-specific survival predicting poor survival in breast cancer [27]. Al Murri et al. reported negative findings of the association of CRP as a prognostic marker of breast cancer [28].

Elevated levels at the time of diagnosis of breast cancer indicate aggressiveness of the tumor. In our case-control study from India, among women with breast cancer, we found a significant correlation of serum hsCRP with stage, size and grade of the tumor, and metastasis [29]. Histologically, inflammation is seen rarely in breast cancer, but infiltration of macrophages in invasive breast cancer increases vascularity of tumor resulting in reduced OS and stimulating recurrence [30]. Solid tumors by stimulating inflammatory response create protumorigenic and proangiogenic microenvironment by inducing DNA damage, which in turn progresses the disease by invasion and metastasis. Proteins involved in the early phase of inflammation may aggravate progression of cancer leading to decreased survival of the patients with breast cancer [31]. Sicking et al. supported a possible link of inflammation with prognosis in node-negative breast cancer patients. Preoperative CRP levels were associated with short DFS and OS independent of established prognostic factors. But on gene expression analysis in a subgroup of 72 patients, they for first time highlighted no relation of circulating CRP with gene expression. Numerous epidemiological prospective studies proved CRP as a well-established independent prognostic marker in breast cancer [32]. Breast cancer survivors with the state of chronic inflammation are at risk of recurrence of the disease as well as metabolic disturbances and CVD. One of the possible explanations for this may be that it is a marker of general health and longevity.

## 6. CRP: As a Biomarker of Cardiotoxicity in Breast Cancer

With tremendous advances in diagnostic and therapeutic modalities in the field of oncology, the outcome of breast cancer has been improved dramatically. But although highly effective, anticancer drugs are associated with higher risk of incident cardiotoxicity. So monitoring of patients receiving cancer therapy for development of subsequent cardiotoxicity is a great challenge. Biomarkers may help to identify patients at high risk before starting therapy as well as during follow-up. Overweight and obese women at the time of diagnosis or weight gain after diagnosis is associated with elevated levels of inflammatory markers. This causes metabolic disturbances increasing the risk for diabetes mellitus, hypertension, and cardiovascular diseases (CVD) among breast cancer survivors. Thomson et al. evaluated presence of metabolic syndrome and CRP levels as cardiovascular risk factors in breast cancer survivors on adjuvant hormone therapy. They found elevated CRP in 90.5% of the population (mean 5.1 ± 5.3 mg/dL) [33]. Recently HEAL study commented reduced levels of CRP in 741 breast cancer survivors treated with tamoxifen [34].

Ky et al. compared multiple biomarkers which can predict subsequent cardiotoxicity in breast cancer patients treated with doxorubicin, taxanes, and trastuzumab. They reported a lack of association of CRP levels with cardiotoxicity while troponin I and myeloperoxidase can be used as potential markers of incident cardiac dysfunction in their study participants [35].

HER 2 positive breast cancers are more aggressive with poor survival rate. With the incorporation of monoclonal antibody trastuzumab in their management, significantly reduced mortality, recurrence, and metastasis have been observed with improved DFS. But therapy with adjuvant agent trastuzumab may induce cardiotoxicity. So periodic monitoring for assessment of cardiac function is mandatory because early detection can prevent or reverse this complication by withholding the drug and adding cardiac therapy. In one pilot study, a cohort of 54 HER2 positive women with early breast cancer were prospectively monitored with serum brain natriuretic peptides, hsCRP, and troponin I levels and left ventricular ejection fraction (LVEF) during trastuzumab therapy. Findings on statistical analysis suggested an association of normal hsCRP levels with low future risk of decreased LVEF. This study highlighted the promising role of CRP as a cheap, reproducible, easily available biomarker for identification of trastuzumab-induced cardiotoxicity [36].

## 7. Other Inflammatory Markers in Breast Cancer

Extensive research has also been carried out to evaluate other serum inflammatory markers in breast cancer patients. Various elements of inflammation and enzymes of tissue remodeling are involved in different steps of carcinogenesis and metastasis [6]. CRP and serum amyloid A are acute phase reactant proteins. Estimation of plasma cytokines and interleukins is difficult because of short half-life and presence of blocking substances in circulation. IL-6 has an endocrine capacity and it triggers the hepatic synthesis of CRP. Serum IL-6 has been found to be raised in various cancers and associated with progression of the disease and reduced OS. Cyclooxygenase-2 (COX-2) is a local inflammatory marker in contrast to CRP, which is a systemic marker of inflammation. But among all the markers, CRP is the simplest assay with great accuracy and precision than any other markers [37]. Reliable assays for CRP are widely available with temporal stability. A number of studies assessed multiple inflammatory biomarkers as the prognostic factor in breast cancer. Among them CRP was found to be the most consistent prognostic predictor. Findings of WHEL study reported increased 2-fold risk of all-cause and breast cancer-specific mortality and 67% risk of additional breast cancer-related events in subjects with raised CRP levels during postdiagnosis period [38]. Ravishankaran and Karunanithi determined preoperative levels of serum IL-6 and CRP in breast cancer to correlate them with the stage of the disease and prognosis. They found an association of higher IL-6 with tumor invasion and metastasis with the significant difference in OS. But CRP showed no significance with OS of the patients [39]. Inflammation based prognostic scores are also used to predict the outcome of malignant diseases. As a marker for chronic low-grade inflammation, hsCRP seems to be highly sensitive, cost-effective, easily measurable, and widely available biomarker to predict prognosis of breast cancer patients even in developing setups. It has definitely a positive impact on overall clinical outcomes.

## 8. CRP Lowering Agents in Breast Cancer Therapy

In cardiovascular diseases, CRP lowering drugs like COX inhibitors, platelet aggregation inhibitors, lipid lowering agents, and angiotensin converting enzyme inhibitors and antioxidants have been found to be promising therapeutic targets. Nonsteroidal anti-inflammatory drugs (NSAIDs) are extensively administered COX inhibitors worldwide. These drugs arrest the cell cycle and inhibit the growth of the tumor by inhibiting angiogenesis, neovascularization, and decreasing prostaglandin synthesis. Various clinical trials of NSAIDs showed promising role in reducing the risk of breast cancer [8].

Lipid lowering agent statins also decrease the level of CRP and they have been also found to have an antitumor effect through antiproliferative, antiangiogenic, and antimetastatic properties. But studies have reported inconsistent results for the utility of statin in cancer prevention. The addition of CRP lowering agents along with chemotherapeutic drugs may improve the survival of breast cancer patients.

*Limitations of CRP Assay*. CRP is a sensitive but nonspecific marker of inflammation. Its levels are easily influenced by various physiological and pathological factors like acute and chronic infections and use of anti-infectious agents and anti-inflammatory drugs. Single estimation of CRP level does not help; at least two measurements are needed at the interval of 2 weeks to interpret the risk.

In the present review, we intended to evaluate the role of CRP in every aspect of breast cancer, from its usefulness in risk prediction, diagnosis, prognosis, and therapeutics. The microenvironment of tumors rich in inflammatory cells is an essential element in carcinogenesis. Cancer-related inflammation has emerged as an important hallmark of cancer. Chronic inflammation is also an essential player in recurrence of the disease by the promotion of dissemination and growth of metastatic seeds [40]. CRP, an acute phase reactant nonspecific inflammatory protein, is synthesized by hepatocytes in response to IL-6 mainly. With improved sensitive assays for hsCRP, a number of studies documented its role in breast cancer as a risk marker and prognostic predictor and to identify the risk of cardiac dysfunction among breast cancer survivors.

## 9. Conclusion

Chronic inflammation is a key contributor for breast cancer, right from its causation, initiation, promotion, progression, metastasis, and clinical features. Serum CRP assay is a simple, cheap, and sensitive test available widely. The potential utility of CRP as a risk predictor for breast cancer is questionable in clinical practice. But CRP will have the promising role as an additional prognostic predictor of survival. Also, it is a risk predictor for subsequent cardiotoxicity in patients receiving chemotherapy.

Future studies are needed to decide strategy of implementation of anti-inflammatory CRP lowering agents. This may help in prevention, control of breast cancer, and improving outcome in patients with breast cancer. This offers a hope that overall morbidity and mortality can be minimized to a large extent with improved strategies in the management of breast cancer and appropriate interventions aiming towards better patient care.
